# Comparative Analysis of Nkx2.1 and Islet-1 Expression in Urodele Amphibians and Lungfishes Highlights the Pattern of Forebrain Organization in Early Tetrapods

**DOI:** 10.3389/fnana.2018.00042

**Published:** 2018-05-18

**Authors:** Nerea Moreno, Jesús M. López, Ruth Morona, Daniel Lozano, Sara Jiménez, Agustín González

**Affiliations:** Department of Cell Biology, Faculty of Biology, Complutense University of Madrid, Madrid, Spain

**Keywords:** pallium, subpallium, basal ganglia, amygdala, hypothalamus, homology, evolution

## Abstract

Expression patterns of Nkx2.1 and Islet-1 (Isl1), which encode transcription factors that are key in the regionalization of the forebrain, were analyzed by combined immunohistochemical methods in young adult specimens of two lungfishes (*Neoceratodus forsteri* and *Protopterus dolloi*) and a urodele amphibian* (Pleurodeles waltl)*. We aimed to get insights into the possible organization of the forebrain in the common ancestor of all tetrapods because of the pivotal phylogenetic significance of these two groups, being lungfishes the closest living relatives of tetrapods, and representing urodeles a model of simple brain organization with most shared features with amniotes. These transcription factors display regionally restricted expression domains in adult (juvenile) brains that are best interpreted according to the current prosomeric model. The regional patterns observed serve to identify regions and compare between the three species studied, and with previous data reported mainly for amniotes. We corroborate that Nkx2.1 and Isl1 expressions have very similar topologies in the forebrain. Common features in all sarcopterygians (lungfishes and tetrapods) have been observed, such as the Isl1 expression in most striatal neurons, whereas Nkx2.1 is restricted to migrated interneurons that reach the ventral pallium (VP). In the pallidal derivatives, the combination of both markers allows the identification of the boundaries between the ventral septum, the bed nucleus of the stria terminalis (BST) and the preoptic commissural region. In addition, the high Isl1 expression in the central amygdala (CeA), its boundary with the lateral amygdala (LA), and the scattered Nkx2.1 expression in the medial amygdala (MeA) are also shared features. The alar and basal hypothalamic territories, and the prethalamus and posterior tubercle (TP) in the diencephalon, have maintained a common pattern of expression. This regional distribution of Isl1 and Nkx2.1 observed in the forebrain of urodeles and lungfishes contributes further to our understanding of the first terrestrial vertebrates and their ancestors.

## Introduction

Developmental processes occurring from the neural plate stages entail drastic morphological changes and the topological arrangement of the forebrain subdivisions is a consequence of the location of their primordia in the neural plate. This organization is driven by the combination of basic genetic codes, which are strongly conserved in the different animal models, and the mechanisms leading to the formation and patterning of the brain are highly conserved in evolution. This is specially clear when the forebrain organization of different vertebrates is interpreted according to the prosomeric model based on detailed comparison of diverse developmental gene expression patterns (Puelles and Rubenstein, 2003, 2015).

Among vertebrates, not only convergence and divergence features of brain organization in terms of evolution have been reported, but also secondary adaptations of each group that can be related to sophisticated behaviors or ecological adaptations of each model analyzed. However, in spite of the differences and adaptations there is a basic bauplan in the organization of the forebrain that has been inferred in the last years, thanks to the comparative analysis of the expression patterns of regulatory genes in distinct animal models (e.g., Puelles et al., 2000; Bachy et al., 2001; Brox et al., 2003; Alunni et al., 2004; Moreno et al., 2004, 2005; Osorio et al., 2005; Puelles and Ferran, 2012; Rodríguez-Moldes et al., 2017).

Many shared features have been reported in the forebrain organization across tetrapods, and an interesting issue to resolve in evolutionary terms would be to understand the organization of the forebrain of early tetrapods. To this aim, we have selected two representative groups. First the urodeles, because amphibians are the only tetrapod anamniotes and, therefore, lack the innovations that characterize the amniotes (rather than all tetrapods). Of note, the brains of anuran amphibians are substantially more complex than those of urodeles, which show poorly differentiated brain structures probably due to secondary simplification (e.g., Northcutt, 1987; Roth et al., 1992, 1993), which gives the impression that the brains of newts and salamanders are more primitive than their phylogenetic position, as tetrapods, implies (Roth et al., 1992). Second, we analyzed the forebrain of lungfishes, because they are the closest anamniote relatives of amphibians and it is currently believed that all tetrapods evolved from an ancestral lungfish group (Clack, 2002; Daeschler et al., 2006). Actually, thanks to the use of a large dataset recent phylogenomic analyses effectively resolved that lungfishes (Dipnoi) were the sister group of tetrapods (Chen et al., 2012; Irisarri and Meyer, 2016; Irisarri et al., 2017). Extant lungfishes are divided into two orders, Ceratodontiformes and Lepidosireniformes. The first order has one extant species, the Australian *Neoceratodus forsteri*, whereas the second order is represented by the South American genus *Lepidosiren* (one species) and the African genus* Protopterus* (four species). The brains of lungfishes also exhibit substantial variation and although lepidosirenid lungfishes have extremely simple brains that look in many ways like those of urodeles, *Neoceratodus* *forsteri* has more complex brain organization. Actually, in early comparative neuroanatomy the brain of urodeles was chosen to focus on the species with the simplest brains (Herrick, 1948), also because salamanders were easier to obtain and work with than lepidosirenid brains.

In previous studies of forebrain development and organization, the analysis of the expression patterns of the transcription factors Nkx2.1 and Islet-1 (Isl1), which are very conserved in the evolution, has been shown to be an excellent tool to highlight details of telencephalic and hypothalamic regionalization that are readily comparable across species (Sussel et al., 1999; González et al., 2002, 2014; Osorio et al., 2005; Moreno et al., 2008a,b, 2010, 2012b; Moreno and González, 2011; Medina et al., 2014; Santos-Durán et al., 2015, 2016; Sugahara et al., 2016). Isl1 belongs to the family of LIM-hd transcription factors known to exert a variety of functions in the nervous system (Hobert and Westphal, 2000; Sandberg et al., 2016; Magno et al., 2017; Waclaw et al., 2017). In addition, during the last years it has been demonstrated that LIM-hd family members are strongly expressed in highly specific neuromeric domains in the forebrain of all vertebrates analyzed, from lampreys to humans (Rétaux et al., 1999; Bachy et al., 2001; Moreno et al., 2004, 2008a,b, 2010, 2012a; Moreno and González, 2011; González et al., 2014; Medina et al., 2014; Chi et al., 2017). Isl1 is implicated in the general basal forebrain organization: phenotype acquisition, path finding, and neural differentiation (for review, see Hobert and Westphal, 2000; Wang and Liu, 2001; Moreno et al., 2008a,b; Domínguez et al., 2013, 2014; Cho et al., 2014; Herget et al., 2014; Lu et al., 2014; Lee et al., 2016; Waclaw et al., 2017). In the case of Nkx2.1, it is an essential regulator of the medial ganglionic eminence, the preoptic region (POA) and the hypothalamus (Sussel et al., 1999; van den Akker et al., 2008; Sandberg et al., 2016; Magno et al., 2017). Interestingly for our purposes, both Isl1 and Nkx2.1 continue to be expressed in the forebrain after development in a very specific pattern of regionalization.

In the present comparative study conducted to infer how the forebrain of early tetrapods could be organized, we analyzed in detail the patterns of Isl1 and Nkx2.1 expression in the forebrain of *Protopterus dolloi* and *Neoceratodus forsteri*, as representatives of the two extant orders of lungfishes, and the urodele *Pleurodeles waltl*. Single and combined immunohistochemical approaches have allowed the identification of precise subdivisions and boundaries in the telencephalon and hypothalamus that are consistent with the current prosomeric model of forebrain regionalization.

## Materials and Methods

### Animals and Tissue Preparation

For the present study, juveniles of the urodele amphibian *Pleurodeles waltl* (*n* = 11) and the lungfishes *Protopterus dolloi* (*n* = 9) and* Neoceratodus fosteri* (*n* = 6) were used. The specimens of the urodele were obtained from laboratory stock in the Department of Cell Biology, University Complutense, Madrid. The African lungfish (*P. dolloi*) were purchased from commercial suppliers (PezyCia, Madrid, Spain), and the Australian lungfish (*N. forsteri*) were obtained from Jindalee International Pty Limited in Milton, Queensland, an approved breeder and exporter. The original research reported herein was performed according to the regulations and laws established by the European Union (2010/63/EU) and Spain (Royal Decree 53/2013) after approval from the Complutense University to conduct the experiments described. In addition, the *N. forsteri* specimens were handled by Dr. Glenn R. Northcutt (Scripps Institution of Oceanography and Department of Neurosciences, School of Medicine, University of California, San Diego, La Jolla, CA, USA) and the initial steps of perfusion and fixation were conducted in the USA (the brains were then shipped to Spain) following the standards established by the Institutional Animal Care and Use Committee at the University of California, San Diego, for the care and handling of animals in research.

The animals were deeply anesthetized by immersion in 0.01% tricaine methanesulfonate solution (MS222, Sandoz Basel, SW; pH 7.3) and perfused transcardially with physiological saline followed by 200 ml of cold 4% paraformaldehyde in a 0.1 M phosphate buffer (PB, pH 7.4), or MEMFA (0.1 M MOPS [4-morpholinopropanesulfonic acid], 2 mM ethylene glycol tetraacetic acid, 1 mM MgSO_4_, 3.7% formaldehyde). The brains were removed and kept in the same fixative for 2–3 h. Subsequently, they were immersed in a solution of 30% sucrose in PB for 4–6 h at 4°C until they sank, then embedded in a solution of 20% gelatin with 30% sucrose in PB, and stored for 6 h in a 3.7% formaldehyde solution at 4°C. The brains were cut on a freezing microtome at 30–40 μm in the transverse or sagittal plane, and sections were collected and rinsed in cold PB.

### Immunohistochemistry

Single immunodetections of Isl1 or Nkx2.1 were carried out on the free-floating sections, as follows: (1) first incubation was conducted for 48 h at 4°C in the dilution of each primary antibody: mouse anti-Isl1 primary antibody (1:500; Developmental Studies Hybridoma Bank (DSHB), Iowa City, IA, USA. Clone: 39.4D5) or rabbit anti-Nkx2.1 (1:500; Biopat Caserta, Italy. Code number: PA 0100); and (2) second incubations were for 90 min at room temperature with either Alexa 488-conjugated goat anti-mouse (1:500, Molecular Probes, Eugene, OR; catalog reference A21042) or Alexa 594-conjugated goat anti-rabbit (1:500, Molecular Probes; catalog reference A11037).

To study the relative distribution of the two markers in the same sections, the two-step protocol for immunofluorescence was used with cocktails of pairs of primary and secondary antibodies, at the same dilutions and conditions previously specified: (1) first incubation for 48 h at 4°C in the cocktail mouse anti-Isl1/rabbit anti-Nkx2.1; (2) second incubation for 90 min at room temperature in the cocktail Alexa 488-conjugated goat anti-mouse/Alexa 594-conjugated goat anti-rabbit.

In all cases, antibodies were diluted in PB containing 0.5% Triton X-100. After the immunohistochemical steps, the sections were rinsed and mounted on glass slides, which were coverslipped with fluorescence mounting medium, containing 1.5 μg/ml 4′,6-diamidino-2-phenylindole (DAPI) for DNA counterstaining (Santa Cruz; SC-24941 or Vectashield H-1500).

### Controls and Specificity of the Antibodies

The antibodies were previously tested in the species used in this study and many of them were used as territory markers in the forebrain (Moreno and González, 2007a, 2011; González and Northcutt, 2009; González et al., 2014). General controls for the immunohistochemical reaction included: (1) controls in which either the primary antibody or the secondary antibody was omitted; (2) pre-absorption of the primary antibodies with synthetic peptides. The latter included absorption with Isl1 peptide (Abcam, Cambridge; at 0.1, 1.0, or 10.0 μM); (2) and synthetic NKX2.1 peptide (Biopat; 0100-P; at 0.1, 1.0, or 10.0 μM). In all controls, the immunostaining was eliminated.

### Evaluation and Presentation of the Results

**Figure 1:**
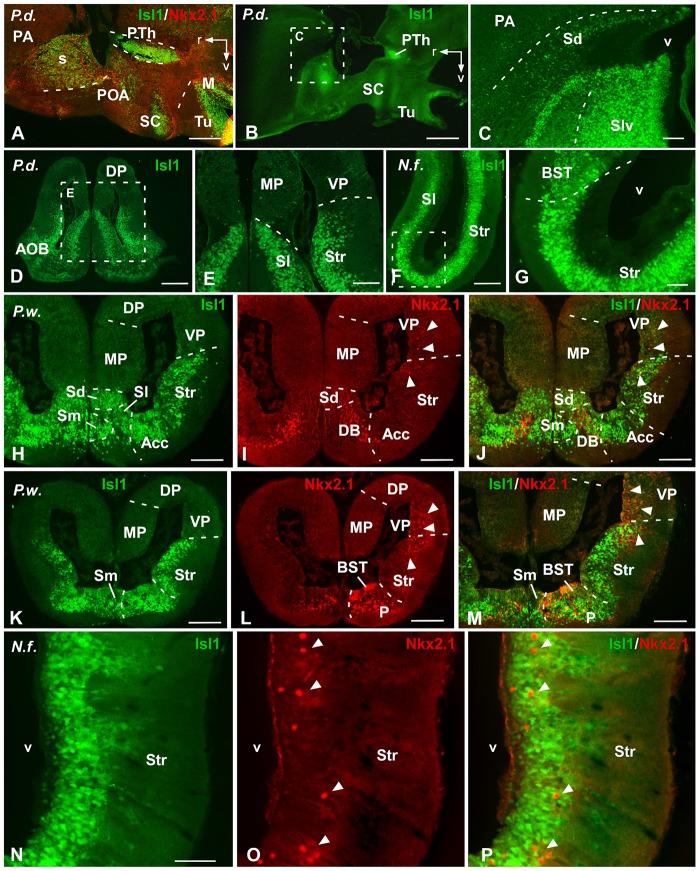
Comparative Isl1/Nkx2.1 expression in the telencephalon. Photomicrographs of sagittal **(A–C)** and transverse **(D–P)** sections through the telencephalon of *Protopterus dolloi* **(A–E)**, *Neoceratodus forsteri* **(F,G,N–P)** and *Pleurodeles waltl* **(H–M)**. In the telencephalon, the combined immunohistochemical detection of Isl1 (green) and Nkx2.1 (red), expressed in the subpallium, clearly allowed the identification of the boundaries of this region and the identification of the marked areas. Arrowheads point to Nkx2.1 labeled cells in striatal and ventral pallial areas. Scale bars = 500 μm **(A,B,D,K–M)** and 200 μm **(C,E–G,H–J,N–P)**. See list for abbreviations.

**Figure 2:**
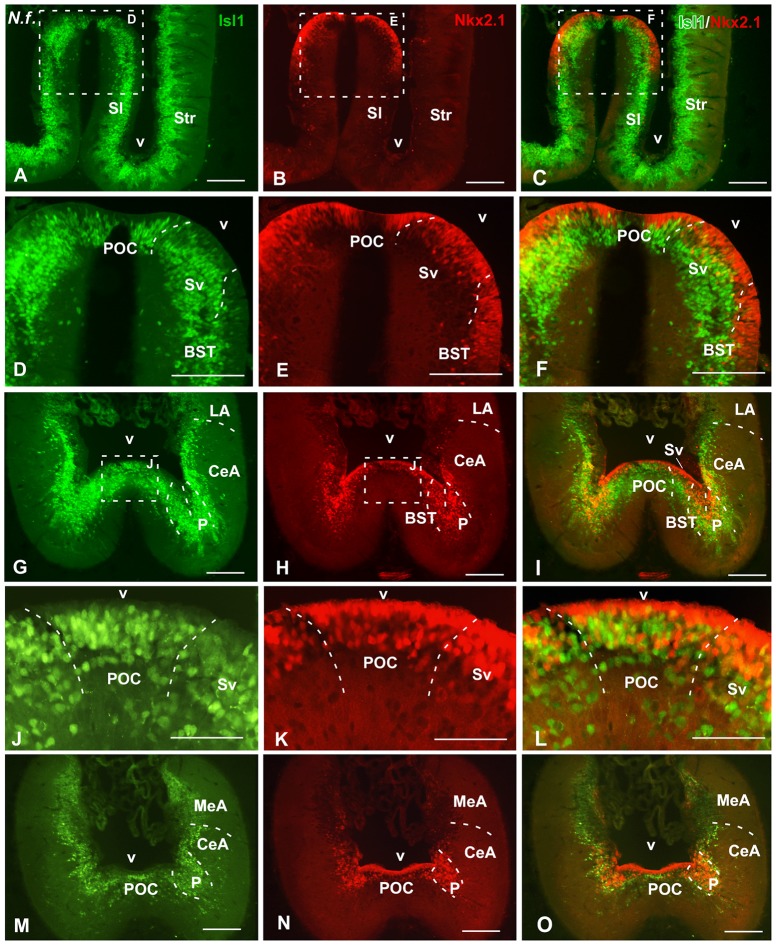
Isl1/Nkx2.1 expression in the subpallium of *Neoceratodus forsteri*. Photomicrographs of transverse sections through the caudal telencephalon of *Neoceratodus forsteri*, ordered from rostral **(A–C)** to caudal **(M–O)**. In the subpallial part of the telencephalon, the combined immunohistochemical detection of Isl1 (green) and Nkx2.1 (red), allowed the identification of the boundaries of this region and the codistribution of both markers in septal, pallidal, striatal and amygdaloid regions. Note the distribution of Nkx2.1 cells in ventricular and mantle zones, whereas the Isl1 labeling is restricted to cells in the mantle. Scale bars = 200 μm **(A–I,M–O)** and 100 μm **(J–L)**. See list for abbreviations.

**Figure 3:**
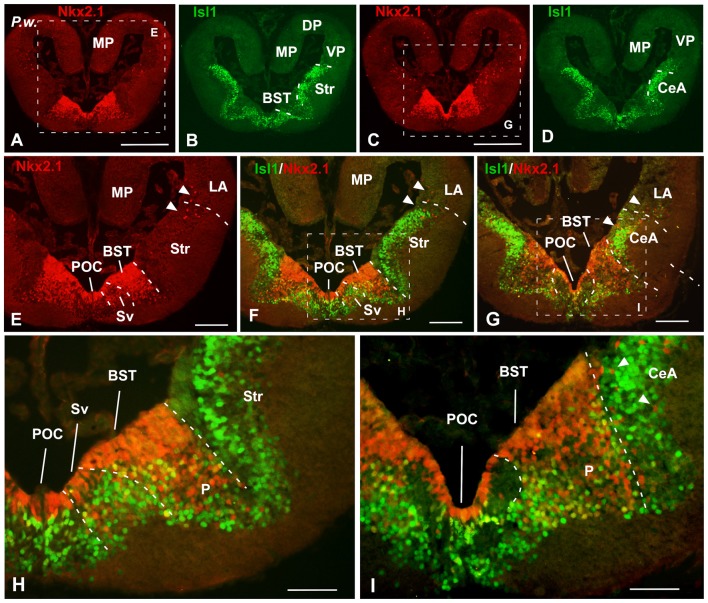
Isl1/Nkx2.1 expression in the subpallium of *Pleurodeles waltl*. Photomicrographs of transverse sections through the telencephalon of *Pleurodeles waltl* at caudal levels of the interhemispheric connection of the lateral ventricles. In the subpallial part of the telencephalon, the combined immunohistochemical detection of Isl1 (green) and Nkx2.1 (red), allowed the identification of the boundaries of this region and the identification of the marked areas. Note the distribution of Nkx2.1 cells in ventricular and mantle zones, in the ventromedial region, whereas the Isl1 labeling is restricted to cells in the mantle of the lateral and medial parts of the subpallium. Arrowheads point to Nkx2.1 labeled cells in striatal, central amygdala (CeA) and ventral pallial areas. Scale bars = 500 μm **(A–D)**, 200 μm **(E–G)** and 100 μm **(H,I)**. See list for abbreviations.

**Figure 4:**
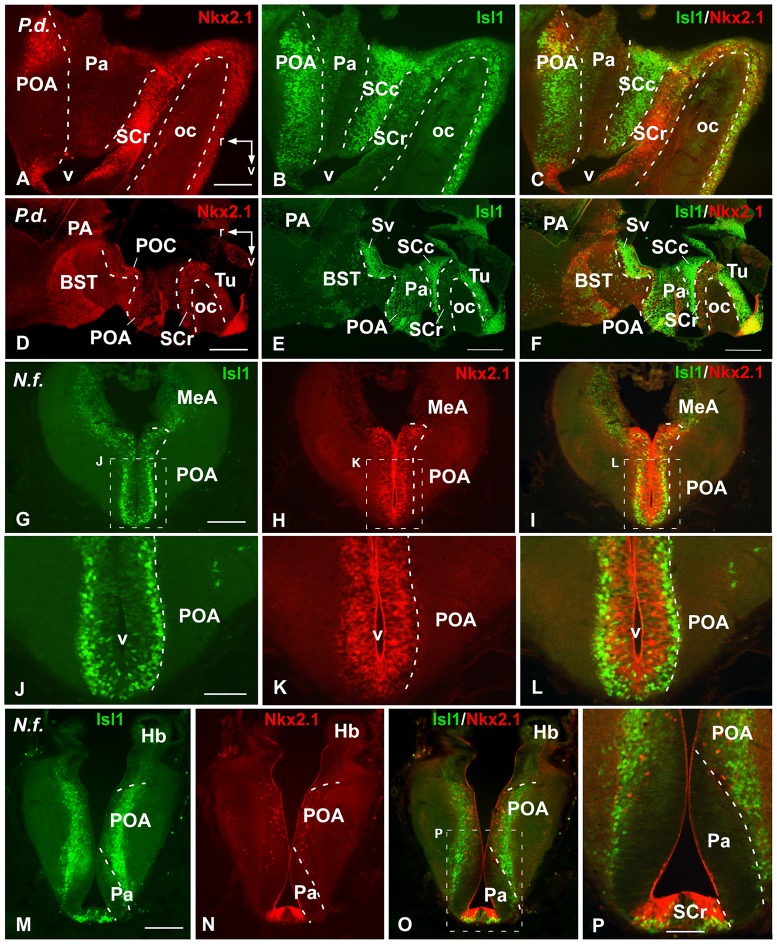
Comparative Isl1/Nkx2.1 expression in the lungfish preoptic and adjacent areas. Photomicrographs of sagittal sections of *Protopterus dolloi* **(A–F)** illustrating preoptic and hypothalamic areas, and transverse sections of *Neoceratodus forsteri* **(G–P)** through the caudal telencephalon. In the preoptic and hypothalamic regions, the banded pattern of expression is clearly seen in the sagittal sections **(A–F)**. Note that the Isl1/Nkx2.1 expressions in the preoptic area (POA) is distinctly located in cells close to the ventricle (Nkx2.1) and more separate in the mantle (Isl1) **(G–L)**. While Isl1 intensely labels the caudal POA and the ventral part of the suprachiasmatic region, Nkx2.1 scattered cells are distributed in the POA and intense labeling is found in the rostral suprachiasmatic nucleus (SC), mainly in the ventricular lining **(M–P)**. Scale bars = 200 μm **(A–C,G–I,M–O)**, and 500 μm **(D–F)** and 100 mm **(J–L,P)**. See list for abbreviations.

**Figure 5:**
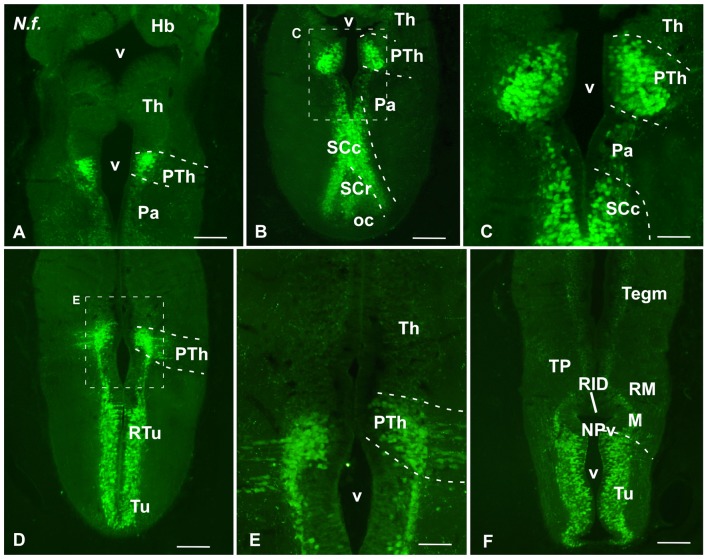
Isl1 expression in the diencephalon and hypothalamus of *Neoceratodus forsteri*. Photomicrographs of transverse sections through the secondary prosencephalon of *Neoceratodus forsteri*. In this region Isl1 is exclusively found in the prethalamic portion of the diencephalon **(A–E)**, whereas in the hypothalamus it avoids the paraventricular area and it is mainly found in the caudal portion of the suprachiasmatic region, with reduced expression in its rostral part **(A,B)**. Isl1 is markedly expressed in the tuberal and retrotuberal hypothalamus **(D,F)**. Scale bars = 500 μm **(A,B,D)**, 200 μm **(E,F)** and 100 μm **(C)** See list for abbreviations.

**Figure 6:**
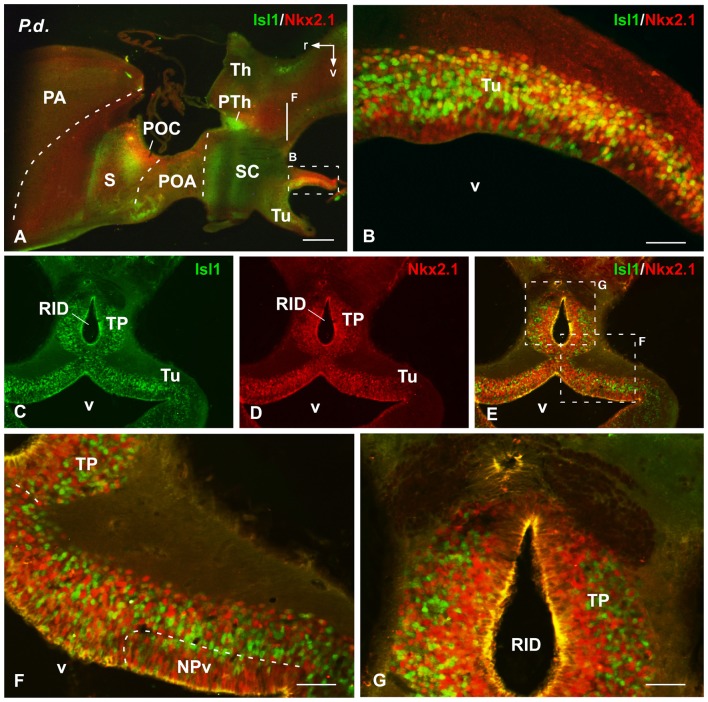
Isl1/Nkx2.1 expression in the hypothalamus of *Protopterus dolloi*. Photomicrographs of sagittal **(A,B)** and transverse **(C–G)** sections through the hypothalamus of *Protopterus dolloi*. The cells stained for Isl1 and Nkx2.1 in the basal hypothalamus are largely intermingled and extend into the posterior tubercle (TP), which in this species shows a dorsal ventricular recess (RID). Scale bars = 500 μm **(A–F)**, 100 **(B)** and 200 μm **(G–I)**. See list for abbreviation.

**Figure 7:**
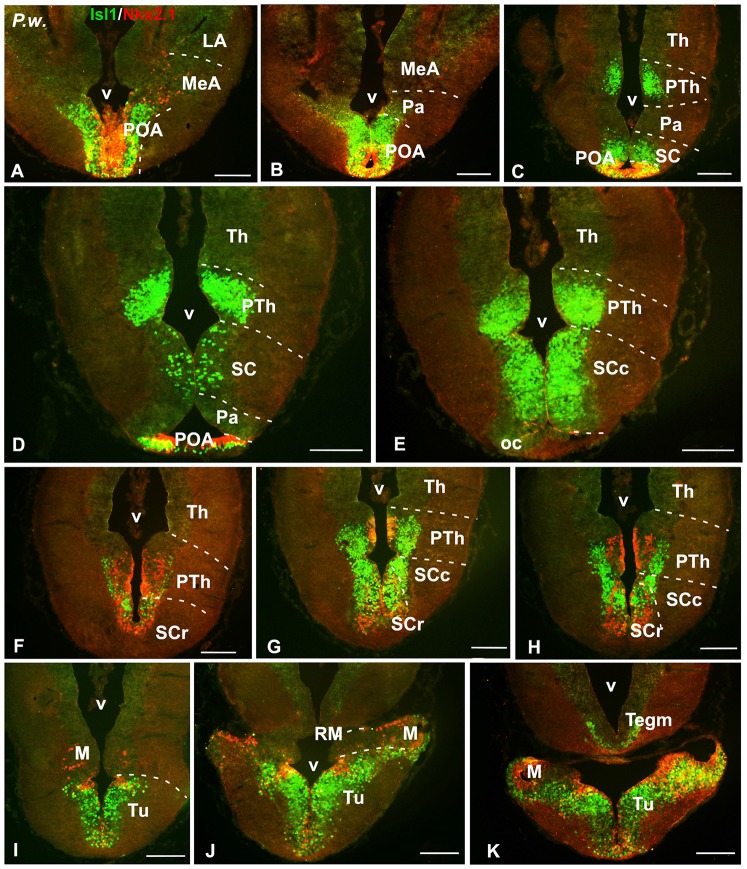
Isl1/Nkx2.1 expression in the diencephalon and hypothalamus of *Pleurodeles waltl*. Photomicrographs of transverse sections through the diencephalon and hypothalamus of *Pleurodeles waltl*, organized from rostral **(A)** to caudal **(K)**. Isl1 and Nkx2.1 are intensely expressed from the POA **(A,B)**, and Isl1 in the subparaventricular region containing the SC, mainly in its caudal subdivision **(C,H)**. Nkx2.1 is mainly confined to the rostral part of the SC **(F–H)**. In the basal hypothalamus, both markers are found in the tuberal part and practically only Nkx2.1 cells extend into the mamillary region **(I–K)**. In the diencephalon, Isl1 is abundantly expressed in the alar prethalamus **(C–E)**, whereas in the basal part both markers are found, being the Isl1 cells more laterally located than the Nkx2.1 cells **(F–H)**. Scale bars = 200 μm. See list for abbreviations.

The sections were analyzed with an Olympus BX51 microscope equipped for fluorescence with appropriate filter combinations. Selected sections were photographed with a digital camera (Olympus DP72) and representative photomicrographs of brain regions with significant labeling were taken and presented in Figures 1–7. Contrast and brightness were adjusted in Adobe Photoshop CS3 (Adobe Systems, San Jose, CA, USA), and photomicrographs were mounted on figures in Canvas 11 (ACS Systems International).

The nomenclature used is essentially the same followed in previous recent studies of urodele and lungfish brains (e.g., González and Northcutt, 2009; Joven et al., 2013a,b; González et al., 2017; López and González, 2017). Of note, we followed the neuromeric (segmental) model of brain organization currently used for the regionalization of the different parts of the central nervous system of vertebrates (Puelles and Rubenstein, 2003, 2015).

## Results

The distribution of the cells containing the Isl1 and Nkx2.1 proteins were analyzed by immunohistochemical methods in the three species, focusing mainly on their localization in particular regions of the forebrain. The sections with double staining served to evaluate the precise codistribution of both markers, which allowed us the identification of landmarks and boundaries between expressing and non-expressing territories, identified and named according to previous reports in other vertebrates using the same markers (e.g., Moreno et al., 2008a, 2010). In the following descriptions, we will deal with the main regions of the forebrain comparatively, and major traits of expression will be detailed for the urodele and the lungfish species. In the case of lungfishes, two different species have been used and the results were, in general terms, very similar. Therefore, in the description of the results, the specific mention of a particular species is only made when differences were observed, trying to avoid repetitions.

### Telencephalon

In the telencephalon both in the urodele and in the lungfishes, Isl1 and Nkx2.1 were never expressed in the pallium and the olfactory bulbs, but in contrast, both markers were intensely and distinctly expressed in the subpallium (Figures 1–3).

**Figure 8:**
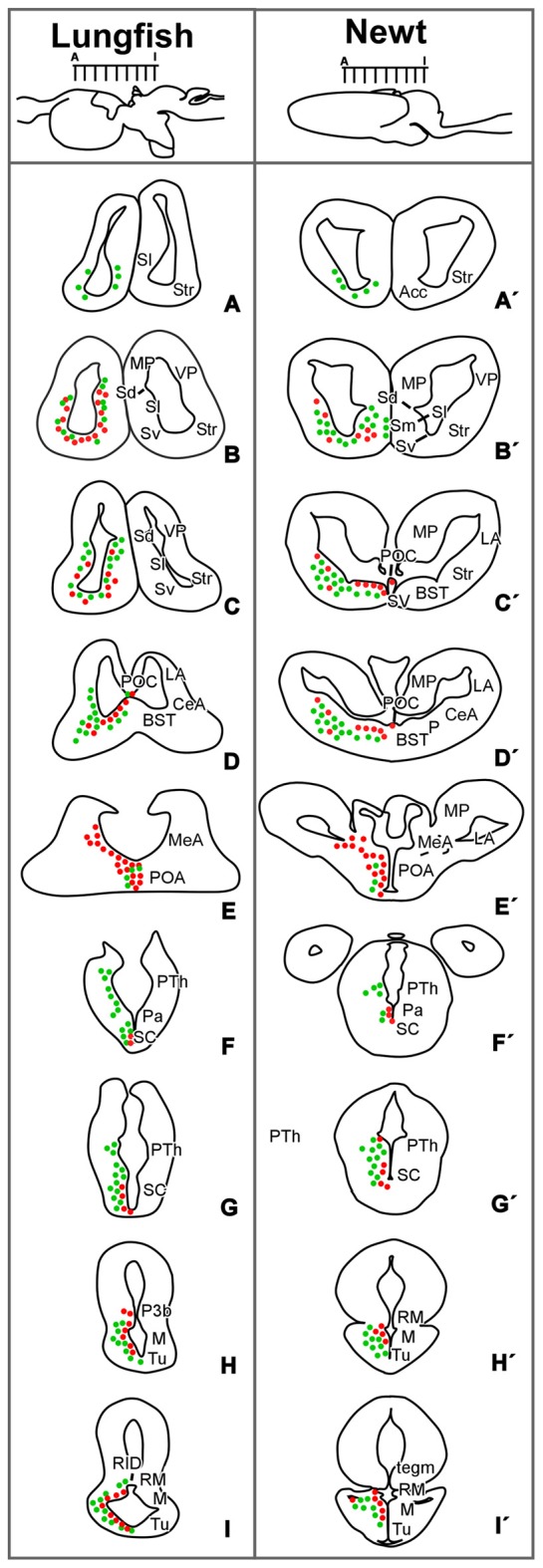
Comparative drawings for the distribution of Isl1 and Nkx2.1 cells. Diagrams of transverse sections through the brain of *Protopterus dolloi* **(A–I)** and *Pleurodeles waltl* **(A′–I′)** illustrating the distribution of Isl1/Nkx2.1-expressing cells (green/red dots) at the rostrocaudal levels indicated in the lateral view of the brain.

At mid-rostral regions of the telencephalon of both lungfishes the labeling for Isl1 was particularly abundant in the striatal and septal regions (Figures 1D–G). In the striatum, abundant migrated cells expressing Isl1 were observed in dorsal and ventral regions. At these levels, the Isl1 labeling highlighted a large septal region that ascended dorsally in the medial telencephalic wall, where the expressing cells were concentrated in the lateral septum (Figures 1E,F). Of note, at these rostral and mid telencephalic levels Nkx2.1 expression was not detected (see Figure 8A). In the case of the amphibian *Pleurodeles*, Isl1 conspicuously labeled cells in the striatal and septal regions (Figures 1H–M). Of note, the Isl1 expressing cells occupied almost completely the septal region of the urodele, where from rostral regions can be found Isl1 cells in the dorsal, medial and lateral septal areas (Figure 1H). The most rostral Nkx2.1 expression was found in the diagonal band, but only in the mantle zone (mz), whereas the vz was devoid of Nkx2.1 expression (Figure 1I). At this telencephalic levels but also caudally, when double labeling was accomplished, scattered Nkx2.1 expressing cells were found in striatal regions among the Isl1 cells (see arrowheads in Figures 1H–P). But only in the case of the amphibian, Nkx2.1 cells were clearly observed dorsal to the Isl1 expressing regions, within the ventral pallium (VP; Figures 1I,J,L,M).

At caudal telencephalic level, distinct staining was observed in the telencephalon impar (Figures 2A–C, 3A–D). In lunghfishes, the vz of the ventral portion of the septum (Sv) was defined by the Nkx2.1 expression (Figures 2A,B,E), whereas cells containing Isl1 were located separated from the ventricle in the mz. In the case of *Pleurodeles*, the double staining highlighted the expression of Nkx2.1 in the Sv, including the vz in contrast to the Isl1 expression observed in this region only in migrated cells in the mz (Figures 3F,H).

In the case of the pallidum in *Pleurodeles*, the cells were migrated ventrolaterally toward a reach a caudoventral location with respect to the striatum (Figures 1L, 3H,I). In lungfishes, the caudal ventrolateral region containing dispersed Nkx2.1 cells was defined as the pallidum (Figures 2H,I,N,O). In both cases, the bed nucleus of the stria terminalis (BST) was characterized by the Nkx2.1 expression in virtually all cells of the vz (Figures 2E,H, 3E). On the contrary, the Isl1 expression in the BST was mainly concentrated in the external portion, avoiding the vz and its proximity. In the case of the amygdaloid region at these levels, the lack of Isl1 and Nkx2.1 expression dorsal to the striatum defined the lateral amygdala (LA) in lungfish and *Pleurodeles* (Figures 2G–I, 3E–G).

In the most caudal portion of the septum, just above the anterior commissure, a particular region was identified as the commissural preoptic area (POC), located medial to the BST (Figures 2E,H,K, 3H,I). In the POC the Nkx2.1 was almost exclusively located in the vz where virtually all cells were Nkx2.1 immunoreactive, whereas the Isl1 expression was restricted to scarce cells out of the vz (Figures 2D–O, 3E–I). At the same caudal levels in the lateral telencephalic wall, the striatum and its caudal continuation, the CeA, were labeled with Isl1 (Figures 2G–I,M,O, 3G,I). Based on the pattern observed with the staining combination, the boundary with the adjacent BST was inferred. Thus, in the striatal and central amygdaloid regions the Isl1 staining was more extensive and almost all cells out of the vz seemed to express Isl1, whereas in the adjacent BST the vz was delineated by the Nkx2.1 expressing cells (Figures 2G–I, 3H,I). Nkx2.1 expression in these lateral regions was found in scattered cells throughout the striatum and the CeA (see arrowheads in Figures 1N,P, 3I). Additionally for the amygdaloid complex, moderate numbers of Isl1 expressing cells were detected in the medial amygdala (MeA), and only scarce and scattered Nkx2.1 cells (Figures 2M–O, 4G–I, 7A,B).

Finally, the preoptic area (POA) in both animal models was defined by the vz expression of Nkx2.1, whereas Isl1 was restricted to the adjacent mantle zone (Figures 4G–L, 7A–C).

### Hypothalamus and Diencephalon

Ventrally adjacent to the POA, in both models the paraventricular area was defined by the absence of Nkx2.1/Isl1 staining, as compared to the adjacent regions (Figures 4A–C, 7C,D). But it is noteworthy that in this hypothalamic territory of *Neoceratodus* Isl1 expressing cells were found scattered in the outermost portion, probably arising from adjacent territories (Figures 5A–C).

In the subparaventricular area, the suprachiasmatic region was defined by the Nkx2.1/Isl1 expression suprachiasmatic nucleus (SC; Figures 4A–C, 5B,C, 7D–H). In this zone, caudal and rostral regions were discerned based on the Nkx2.1/Isl1 combination. The caudal subdivision was rich in Isl1 labeled cells adjacent to the paraventricular area devoid of both Isl1 and Nkx2.1 expression. In addition, the rostral subdivision showed the Nkx2.1 expression in the vz, surrounded by Isl1 expressing cells (Figures 4A–C,P, 5B,C, 7E–H).

Within the basal hypothalamus, the combination of both markers allowed the identification of the boundary between the tuberal region where both markers were expressed (Figures 5D–F, 6A–E, 7I–K) and the mamillary portion, which was devoid of Isl1 expression (Figures 5F, 7I,K). In the case of the lungfishes, a pronounced ventricular recess exists in the caudally located posterior tubercle (TP; dorsal infundibular recess, RID) where the abundant cells labeled for both markers intermingled (Figures 6C–G). In the caudal tuberal hypothalamus of both urodeles and lungfishes, lies the prominent nucleus of the periventricular organ (NPv), which in lungfishes forms a band that extends along the infundibulum and showed primarily Nkx2.1 labeled cells (NPv in Figures 5F, 6F).

Additionally, distinct and intense expression of Isl1 was observed within the prethalamus of the three species studied (Figures 1A,B, 5A–E, 7C–H). The Isl1 labeling did not include the cells in the ventricular lining and, abundant Isl1 positive cells were observed at caudal levels into more lateral positions (Figures 5D,E). The expression of Nkx2.1 was localized in cells intermingled with the Isl1 cells in the basal part of p3 (Figures 6C–G). In the case of the urodele Nkx2.1 expression was detected in the ventricular zone of the ventral part of p3 (Figures 7G,H).

## Discussion

### Early Tetrapod Forebrain Regionalization Based on Isl1 and Nkx2.1 Expression

**Figure 9:**
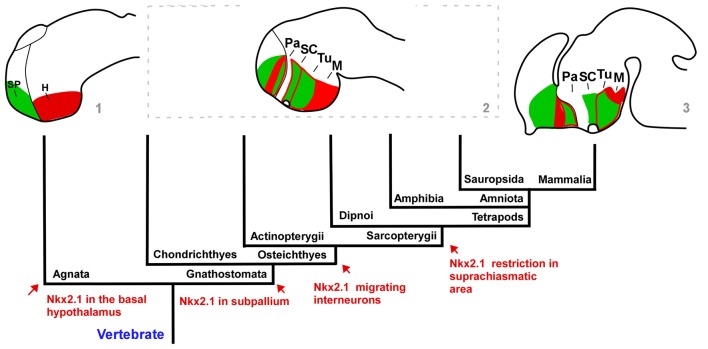
Cladogram representing the regionalization of the prosencephalon based on Isl1 (green)/Nkx2.1 (red) expression. The distribution of the two markers used in our study of the lungfish and urodele forebrains are compared with the situation described in other vertebrate groups such as the lamprey (agnathan) and mouse in which the major differences are found. In all groups, the regions analyzed mainly include comparable molecular compartments. Regarding the Nkx2.1 expression, the main evolutionary events and the moment in which they appear are mentioned and indicated in the scheme by arrows. Note that the developmental stages used in the brain schemes are not equivalent. The schematic representations are made based on the data from: (1) Murakami et al. (2001) and Osorio et al. (2005); (2) present results; Herget et al. (2014), Domínguez et al. (2015) and Santos-Durán et al. (2015); and (3) Puelles et al. (2012), indicated in the figure by the numbers.

On the basis of shared patterns of expression for the two transcription factors analyzed, we discuss the condition that characterize distinct forebrain regions across tetrapods and that most likely existed in their common ancestor (Figures 8, 9).

#### Telencephalon

In the medial wall of the telencephalic hemispheres, the septum is greatly reduced in urodeles (Northcutt and Kicliter, 1980; González et al., 2017) and the precise extension of the septal region in lungfishes has been a very controversial subject in previous neuroanatomical studies (Nieuwenhuys and Meek, 1990; Nieuwenhuys, 1998). First, there are major differences in the hemispheres in *Protopterus* and *Neoceratodus* and the structures in the medial wall located ventral to the ependymal membrane in *Neoceratodus*, and most of the thick medial wall of *Protopterus* have been believed by some researchers to be homologous to the septum in other vertebrates, proposing therefore that the medial pallium (MP) of this animals is a very reduced structure (Nieuwenhuys and Meek, 1990; von Bartheld et al., 1990; Nieuwenhuys, 1998). In contrast to this “*restricted pallium hypothesis*” other authors proposed the “*extended pallium hypothesis*” in which the medial telencephalic wall in lungfishes is constituted by a dorsal MP and a ventral septum (Northcutt, 1984; Reiner and Northcutt, 1987) as in other vertebrates. This latter hypothesis has been particularly reinforced by different immunohistochemical studies (see López et al., 2017b), and the combination of Nkx2.1 and Isl1 that allow the septal identification from rostral to caudal levels supports it (present results). Thus, based on Isl1 the lungfish septum has a large lateral septum and a small medial septum, located in the most medial wall, whereas caudally the ventral septum was delineated based on Nkx2.1 expression. In addition, in both, urodeles and lungfishes the ventral boundary of the septum with the BST is identified based on the particular expression of Nkx2.1 in the ventricular zone (present results), as previously established in *Xenopus* (Moreno et al., 2008a, 2012b) and amniotes (Puelles et al., 2000). In anurans, the septum is particularly enlarged (Northcutt and Kicliter, 1980; González et al., 2017) and the Isl1 expression, and specially its combination with Nkx2.1, highlights differences between the lateral and the ventral portions (rich in Isl1 cells), the medial and dorsal portions (scarce Isl1 cells), and the BST (virtually devoid of Isl1 cells; Moreno et al., 2008a). In lughfishes and *Pleurodeles*, very similar observations have been made (present results).

In the case of the basal ganglia, the striatum of urodeles and lungfishes occupies the ventrolateral part of the hemispheres but the existence and precise localization of the pallidum has been poorly established and questioned (see Marín et al., 1998a; González et al., 2017; López et al., 2017b). In this respect, to interpret the components of the basal ganglia the labeling of Isl1 and Nkx2.1 is crucial (present results), as confirmed for most vertebrates studied (Puelles et al., 2000; Moreno and González, 2011; González et al., 2014). The striatum derives from the embryonic lateral ganglionic eminence (LGE) and produces all the projection neurons of the striatonigral and striatopallidal systems. Isl1 is required for the survival and differentiation of direct pathway striatonigral neurons during embryonic development, not only by orchestrating survival, differentiation, and axonal projections of striatonigral neurons but also by suppressing striatopallidal-enriched genes (Ehrman et al., 2013; Lu et al., 2014; Waclaw et al., 2017). The dual action of developmental control by Isl1 in promoting appropriate striatonigral but repressing inappropriate striatopallidal genetic profiles may ensure sharpening of the striatonigral identity during development (Lu et al., 2014). Similarly, in the striatum of the urodeles and lungfishes studied, Isl1 shows an outstanding expression, even in the juvenile stages analyzed (present results; Moreno and González, 2007a; González and Northcutt, 2009). In line with these results, a recent study on the localization of the protein DARPP32 (Dopamine and cAMP-Regulated PhospoProtein of relative molecular mass 32,000 daltons), which is particularly expressed in striatal projection neurons, has demonstrated a pattern of expression in the striatum of lungfishes coincident with that for Isl1 (López et al., 2017a). Both in urodeles and lungfishes (present results), the comparison of the Isl1 staining with the dopaminergic projection from the mesencephalic tegmentum, which forms a dense neuropil in the striatum of all tetrapods, reveals that a dorsal striatal territory, just beneath the VP, lacks Isl1 expression. Of note, in other vertebrates the main body of the LGE that will give rise to the striatal projection neurons is the one with intense Isl1 expression but a second small dorsal territory lacks Isl1 cells (Stenman et al., 2003; Moreno et al., 2009) and our observations suggest that it is a highly conserved condition in evolution, shared by all the vertebrates studied.

With respect to the pallidum, it was shown that in birds and mammals Nkx2.1 is early expressed in the ventricular and mantle zones of the medial ganglionic eminence (MGE, primordium of the pallidum; Puelles et al., 2000). It is also expressed in the neighboring caudal part of the septum, and in cells of the diagonal band complex and adjacent POA (Puelles et al., 2000). Results in anuran amphibians and reptiles showed that the Nkx2.1 expression continues after embryonic and larval development in similar ventricular and migrated cells in comparable regions (González et al., 2002; Moreno et al., 2010, 2012b). Similarly, we have observed in this study that the pallidal structures, most likely derived from the homologous region of the MGE, can be discerned in the urodele and lungfish telencephalon on the basis of the ventricular and mantle expression of Nkx2.1, whereas Isl1-expressing cells are located only in the mantle zone and mainly at striatal derivatives. The connections of the basal ganglia in *Pleurodeles* suggested the presence of an actual pallidum ventrocaudal to the Marín et al. (1998b) confirmed by the Nklx2.1 expression (present results). In the case of lungfishes, the connections of the basal ganglia have not been studied, but the region here considered as pallidum coincides with that proposed in *Protopterus* based on the immunohistochemical demonstration of LANT6, a peptide specifically contained in pallidal neurons in amniotes (present results; Reiner and Northcutt, 1987; Moreno and González, 2005).

Of note, in mammals Nkx2.1 is expressed in most striatal interneurons, which derive from the MGE and migrate tangentially to the developing striatum (Sussel et al., 1999; Marín et al., 2000). Both cholinergic and GABAergic subpopulations of Nkx2.1-expressing interneurons have been reported in the mammalian striatum (Marín et al., 2000). Similarly, the striatal primordium in reptiles and birds does not express Nkx2.1 in its ventricular zone (LGE) and a similar tangential migration of Nkx2.1-expressing cells seems to occur from the pallidal primordia to the striatal mantle (Puelles et al., 2000; Métin et al., 2007). Moreover, similar tangential migration of Nkx2.1 expressing cells originated from the MGE to the striatum was demonstrated also in anuran amphibians (Moreno et al., 2008a). Therefore, the scattered Nkx2.1 cells observed in the striatal region of urodeles and lungfishes suggest the existence of interneurons in the striatum that would have migrated tangentially from the MGE (present results). In support of this condition is the coexpression of Nkx2.1 with ChAT (choline acetyltransferase, a cholinergic marker) and NOS (nitric oxide synthase) that exists in striatal neurons (personal observations in lungfishes, data not shown) as in all tetrapods and this makes it tempting to hypothesize that a migration of interneurons from the MGE to the striatum might be also a primitive feature at the base of all sarcopterygians, presently shared by tetrapods and lungfishes (Figure 9).

Interestingly, genetic studies in mammals have shown that development of the bulk of cortical interneurons depends on Nkx2.1 function (Sussel et al., 1999; Pleasure et al., 2000), and experimental embryological analysis has demonstrated that a large population of cortical interneurons derives from the MGE (Sussel et al., 1999; Wichterle et al., 1999; Anderson et al., 2001). However, Nkx2.1 cells are absent from pallial regions in anurans, birds and mammals (Sussel et al., 1999; Marín et al., 2000; Puelles et al., 2000; González et al., 2002), suggesting that cortical interneurons derived from the MGE down-regulate the expression of Nkx2.1 during their tangential migration to the cortex. The presence in the pallium of amphibians and lungfishes of GABAergic, nitrergic and calretinin containing cells postulate them as pallial interneurons (present results; Morona and González, 2008; González and Northcutt, 2009; Morona et al., 2018), and the lack of Nkx2.1 expression, except for the VP in the case of *Pleurodeles* (present results), would also reflect a down-regulation in its expression.

In the case of the amygdaloid complex of *Pleurodeles* and lungfishes, its localization and organization have been a matter of debate from early anatomical studies (see Nieuwenhuys, 1998; ten Donkelaar, 1998). Three components were identified on the basis of chemoarchitecture and scarce hodolgical data, i.e., lateral, central and MeA (Moreno and González, 2007a,b; González and Northcutt, 2009; Northcutt, 2009, 2011; Northcutt and Rink, 2012). In the present analysis based on the Isl1/Nkx2.1 combination, the LA was identidied dorsal to the striatal Isl1 expressing region, within the VP (present results; Figure 8). It has been described that in both groups this area contains abundant nitrergic cells and fibers, like in anurans (Moreno and González, 2007a; González and Northcutt, 2009). Originally, this region in lungfishes was identified as a dorsal striatal territory and it was described as the “intercalated nucleus” (Nieuwenhuys, 1998), however the lack of Isl1 expression demonstrates its striatal nature (present results). Additionally, it has been described that it receives substantial olfactory input (Moreno and González, 2007a; Northcutt and Rink, 2012) like the LA of anurans (Moreno and González, 2006), the ventropallial derivatives of the basolateral components of the amygdala in mammals, or the posterior dorsal ventricular ridge in reptiles (McDonald et al., 1993; Smeets et al., 1997; Marín et al., 1998b; Olmos et al., 2005; Moreno and González, 2006). The striatal component of the complex is defined by the Isl1 massive expression, and as in the rest of vertebrates, it was identified as CeA (present results). In anurans and amniotes, it has long descending projections to the parabrachial area and spinal cord constituting the autonomous portion of the amygdala (Moreno and González, 2005, 2006). The present results prove the origin of this region in *Pleurodeles* and lungfishes, supporting their homological relationships to other vertebrates as well as the common pattern of this region in the evolution. However, further developmental, hodological and functional experiments are needed to characterize this region in the models analyzed. Finally, in the case of the MeA of urodeles and lungfishes, the scarce Isl1 and Nkx2.1 expression (present results) along to its vomeronasal connections and the distinct expression of the homeodomain transcription factor *Orthopedia* (OTP; Moreno and González, 2007a; González et al., 2010; Northcutt and Rink, 2012) has allowed its identification and comparison to its counterparts in anurans amphibians and amniotes (Moreno et al., 2008a; Medina et al., 2017).

As regards the POA, it is located directly in front of the preoptic recess of the third ventricle and it has classically been considered the rostralmost part of the hypothalamus. However, morphogenetic studies of the forebrain have demonstrated that this area develops in the nonevaginated part of the telencephalon (Moreno and González, 2011; Domínguez et al., 2013). The POA in urodeles and lungfishes is defined by the Nkx2.1 vz expression and the Isl1 expression avoiding it (Moreno and González, 2007a; present results). This situation is highly conserved, as described for mammals (Puelles and Rubenstein, 2015), reptiles (Moreno et al., 2012a), *Xenopus* (Moreno et al., 2008a; Domínguez et al., 2013, 2014), or catfish (Santos-Durán et al., 2015, 2016).

#### Hypothalamus

The pattern of labeling in the hypothalamus was analyzed according to the segmental model that clearly establishes that this region is “rostral” to the diencephalon (Domínguez et al., 2015; Puelles and Rubenstein, 2015). Due to the pronounced cephalic flexure of the brain, the rostrocaudal axis is bent almost 90°, and the hypothalamus is topographically located under the diencephalon, as it is observed in classical transverse sections. Therefore, in the current prosomeric model of forebrain organization, the hypothalamus is a component of the secondary prosencephalon located rostral to the diencephalon and ventral to the telencephalon (Puelles and Rubenstein, 2015). The bent alar-basal boundary separates dorsal alar and ventral basal regions within the hypothalamus. The current nomenclature refers to paraventricular and subparaventricular subdivisions in the alar portion, and tuberal and mamillary regions in the basal portion.

The expression patterns of Nkx2.1 and Isl1, among others, have been used frequently to identify and unravel the extent of hypothalamic subdivisions, mainly within the framework of alar and basal regions. Thus, in the alar hypothalamus, the most dorsal portion, called the paraventricular region (Pa), is defined by the lack of Nkx2.1/Isl1, whereas ventrally the SC within the subparaventricular area (SPa) is rich in Isl1 in all the vertebrates analyzed (for review, see Domínguez et al., 2015; Moreno et al., 2017). The situation observed in *Pleurodeles* and the lungfishes is largely similar to those previous results in other vertebrates (present results), strengthening a common pattern in the alar region (Figure 9), which most likely reflects the conservation of its important functional role in the homeostatic regulation. In addition, like in anurans and reptiles the SC is rostrocaudally subdivided based on the Nkx2.1 distinct expression only in the most rostral portion (present results; Moreno et al., 2012a; Domínguez et al., 2013, 2014, 2015). It should be emphasized that the Nkx2.1 is not expressed in the alar hypothalamus of agnathans and chondrichthyes (Santos-Durán et al., 2016), whereas in actinopterygians it seem to be expressed in its whole extent, at least in the zebrafish (Rohr et al., 2001). Through sarcopterygians, it seems that there is a tendency for Nkx2.1 expression to disappear and it is restricted to just a SC subdomain in non-mammalian tetrapods and lungfishes (see Figure 9; van den Akker et al., 2008; Medina and Abellán, 2009; Moreno et al., 2012a; Domínguez et al., 2013, 2015; present results). This situation has been related to the amniote pallial/thalamic expansion- alar hypothalamus reduction (van den Akker et al., 2008; for review, see Medina and Abellán, 2009).

In the case of the basal hypothalamus of lungfishes and *Pleurodeles*, the combination of Isl1 and Nkx2.1 was decisive in the identification of its subdivisions, being both Isl1/Nkx2.1 expressed in the tuberal region of both models (present results), like in all the vertebrates studied (see review in Domínguez et al., 2015). Specially, for example in the case of mammals, it has been described that Isl1 is expressed in several subpopulations of developing arcuate neurons, a nucleus derived from this region (Morales-Delgado et al., 2014) playing crucial roles in their fate specification regulating the gene program that directs development of these feeding- and growth-controlling neurons (Lee et al., 2016). In addition, Isl1 specifies the identity of hypothalamic melanocortin neurons in the mediobasal hypothalamus of all vertebrates (Nasif et al., 2015), whereas Nkx2.1 is found in the terminal and peduncular intermediate portion of the tuberal hypothalamus, which gives rise to the ventromedial and dorsomedial nuclei (Puelles et al., 2012).

The boundary between the tuberal and mamillary territories in the basal hypothalamus is defined in lungfishes and *Pleurodels* by the lack of Isl1 expression in the mamillary region within the continuous Nkx2.1 positive tuberomamillar region (present results). This situation is similar to that observed in anuran amphibians, reptiles and mammals (Puelles et al., 2012; Domínguez et al., 2015; present results). In the case of mammals, the mamillary region only expresses Nkx2.1 (being the caudal and ventral-most portion of the Nkx2.1 domain) while the retromamillary region (RM) expresses Sonic hedgehog (Shh) and other genes in a complementary manner to Nkx2.1 (see Morales-Delgado et al., 2011, 2014; Puelles et al., 2012).

Therefore, despite the different morphology of the hypothalamus in different species, it seems that at least all sarcopterygians share the main features of organization, as evidenced by the common pattern of Nkx2.1 and Isl1 expression, which would surely characterize the condition of their common ancestor.

#### Diencephalon

The two transcription factors analyzed are only expressed in the rostral segment of the diencephalon (p3), in alar (Isl1) and basal (Isl1 and Nkx2.1) regions (see Moreno et al., 2017). The Isl1 expression in the prethalamus is a shared feature among tetrapods and lungfishes (present results; Bardet, 2007; Moreno et al., 2008a). In fact, in mammals LIM-homeodomain genes have been related to the specification of nuclei-specific properties in the diencephalon (Nakagawa and O’Leary, 2001), and particularly Isl1 in the acquisition of the prethalamic dopaminergic phenotype (Andrews et al., 2003). This function might be shared in other vertebrates where similar dopaminergic cells have been demonstrated in the prethalamus (Smeets and González, 2000), and in our particular case in urodeles and lungfishes (González and Smeets, 1991; González et al., 1995; López and González, 2017).

Finally, basal forebrain expression of Nkx2.1 has been demonstrated to extend back to include the p3 basal plate in the mouse (Puelles et al., 2004, 2012; Martínez et al., 2012) and other amniotes (Bardet et al., 2010; Moreno et al., 2017). In agreement with this observation, we have corroborated Nkx2.1 expression in the TP and basal p3 in urodeles and lungfishes, suggesting a shared feature across sarcopterygians.

## Concluding Remarks

In this study, we have conducted a detailed analysis of the regional expression of Isl1 and Nkx2.1, two transcription factors of fundamental relevance in the organization of numerous regions of the forebrain. Although we are aware that these markers do not fully define histological domains, their expressions have been described in homologous regions in different species, and they are useful in the attempt of recognizing particular domains. Given the difficulty for obtaining lungfish embryos, we have taken advance of the fact that the proteins encoded by these two genes are easily detected by immunohistochemistry even after development is completed. Furthermore, since both lungfishes and urodeles show little neuron migration, the localization of the expression is largely comparable to the situation during development.

The regional patterns observed have served to identify regions and compare between the three species studied and with previous data reported mainly for amniotes. We have corroborated that the expression of the genes that encode the transcription factors Nkx2.1 and Isl1 have very similar topologies in their forebrain expression. Thus, major features shared for all sarcopterygians have highlighted the putative primitive condition of the organization of the telencephalon, especially the subpallium. Particularly, in the striatum the Isl1 expression is found in most cells, primarily projection neurons, whereas Nkx2.1 is restricted to migrated interneurons that reach the VP. In the pallidal derivatives, the combination allows the identification of the boundaries between the ventral septum, the BST and the preoptic commissural region. In addition, the high Isl1 expression in the CeA, its dorsal boundary with the LA, and the scattered Nkx2.1 expression in the MeA are also shared features. The alar and basal hypothalamic territories, and the prethalamus and TP in the diencephalon, have maintained a general pattern of expression.

## Author Contributions

All authors had full access to all the data in the study and take responsibility for the integrity of the data and the accuracy of the data analysis. AG and NM devised the study. JL and RM designed and supervised the experiments with lungfishes and urodeles, respectively, and were the primary contributors to the data analysis. SJ and DL performed most of the experiments. NM led the figure preparation and wrote the majority of the article, further completed and edited by AG. All authors approved the article.

## Conflict of Interest Statement

The authors declare that the research was conducted in the absence of any commercial or financial relationships that could be construed as a potential conflict of interest.

## Abbreviations

Acc: accumbens nucleus
AOB: accessory olfactory bulb
BST: bed nucleus of the stria terminalis
CeA: central amygdala
DB: diagonal band of Broca
DP: dorsal pallium
H: hypothalamus
Hb: habenula
LA: lateral amygdala
M: mamillary hypothalamic area
MeA: medial amygdala
MP: medial pallium
NPv: nucleus of the periventricular hypothalamic organ
oc: optic chiasm
P: pallidum
PA: pallium
Pa: paraventricular hypothalamic region
POA: preoptic region
POC: commissural preoptic area
PTh: prethalamus
RID: dorsal infundibular recess
RM: retromamillary region
RTu: retrotuberal region
S: septum
SC: suprachiasmatic nucleus
SCc: caudal part of the suprachiasmatic nucleus
SCr: rostral part of the suprachiasmatic nucleus
Sd: dorsal septum
Sl: lateral septum
Slv: ventral portion of the lateral septum
Sm: medial septum
SP: subpallium
Str: striatum
Sv: ventral septum
Tegm: mesencephalic tegmentum
Th: thalamus
TP: posterior tubercle
Tu: tuberal hypothalamic region
v: ventricle
VP: ventral pallium.
